# Acquired Natural Killer Cell Dysfunction in the Tumor Microenvironment of Classic Hodgkin Lymphoma

**DOI:** 10.3389/fimmu.2018.00267

**Published:** 2018-02-14

**Authors:** Jodi Chiu, Daniel M. Ernst, Armand Keating

**Affiliations:** ^1^Cell Therapy Program, Princess Margaret Cancer Centre, Toronto, ON, Canada

**Keywords:** natural killer cells, Hodgkin disease, tumor microenvironment, immunologic cytotoxicity, killer cell immunoglobulin-like receptor, interleukin-2, immunotherapy

## Abstract

An understanding of interactions within the tumor microenvironment (TME) of classic Hodgkin lymphoma (cHL) has helped pave the way to novel immunotherapies that have enabled dormant and tumor-tolerant immune cells to be reactivated. The immunosuppressive nature of the TME in cHL specifically inhibits the proliferation and activity of natural killer (NK) cells, which contributes to tumor immune-escape mechanisms. This deficiency of NK cells begins at the tumor site and progresses systemically in patients with advanced disease or adverse prognostic factors. Several facets of cHL account for this effect on NK cells. Locally, malignant Reed–Sternberg cells and cells from the TME express ligands for inhibitory receptors on NK cells, including HLA-E, HLA-G, and programmed death-ligand 1. The secretion of chemokines and cytokines, including soluble IL-2 receptor (sCD25), Transforming Growth Factor-β, IL-10, CXCL9, and CXCL10, mediates the systemic immunosuppression. This review also discusses the potential reversibility of quantitative and functional NK cell deficiencies in cHL that are likely to lead to novel treatments.

## Introduction

Despite improvements in the therapy of classic Hodgkin lymphoma (cHL), over 1,000 deaths per year in North America and 10,000 worldwide result from the failure of effective management (1, 2). cHL comprises four histological subtypes and a unique, heterogeneous phenotype (3, 4). Given that 99% of cHL tumor tissue is composed of inflammatory cells (4), the study of the tumor microenvironment (TME) and its interactions with antitumor immune cells has gained increasing relevance. The most promising recent results in patients with relapsed cancers including cHL have come from the use of immunotherapies (5–7), underscoring the notion that tumor-tolerant cytotoxic cells can be reactivated to kill cancer cells (8). Most immunotherapeutic advances against refractory and relapsed cHL have focused on T-cells, while studies with natural killer (NK) cells remain scanty.

Natural killer cells, as a key component of innate anticancer immunity, deserve further investigation in the context of the cHL TME (9, 10). Exploring the interactions between NK cells and cHL TME that drive NK cell-escape mechanisms will provide a better understanding of the targets needed to reverse NK cell anergy, thereby directing treatment strategies.

## Immune Cells in HL TME

While only 1% of tumor tissue is composed of malignant Reed–Sternberg (RS) cells, the remaining 99% of cHL tissue comprises the TME and includes numerous inflammatory cells including B-cells, T-cells [CD4^+^ T-helper cells, regulatory T-cells (Tregs), and cytotoxic CD8^+^ T-cells], macrophages, eosinophils, neutrophils, plasma cells, dendritic cells, and fibroblasts, all meticulously orchestrated by the dysregulated secretion of chemokines and cytokines from both TME and RS cells (11).

Many of the cells, including tumor-tolerant Th2 T-helper cells and Tregs, are recruited for the growth and survival of RS cells (12, 13). Cytokines responsible for the recruitment comprise IL-7, IL-10, Transforming Growth Factor-β (TGF-β), and galectin-1, known to promote tumor expansion, stimulate the differentiation of Tregs, and enhance immunosuppressive interactions between RS cells and cytotoxic T- and NK cells (14–17). Despite the dominance of Tregs and Th2 cells, tumor-antagonizing cells, including NK cells, CD8^+^ T-cells, and Th1 T-helper cells, are still a part of the TME infiltrate. They are attracted by chemokines and cytokines, including CXCL9 (Mig-1), CXCL10 (IP-10), and interferon (IFN)-γ (18–20). These antitumor efforts fail, yielding to tumor-tolerant cells and disease progression (21).

## Importance of NK Cells in the Elimination of Cancer Cells

Natural killer cell effector functions are tightly controlled by the balance between inhibitory and activating signals, as recently reviewed (9, 22). Activating receptors include NKG2D, natural cytotoxicity receptors (NCRs), DNAM1, and Fcγ-RIIIa CD16, among others. Inhibitory signals are mainly mediated by killer cell immunoglobulin-like receptors (KIRs), CD96, and the immune checkpoint receptors, programmed cell death protein-1 (PD-1), T-cell immunoreceptor with Ig and ITIM domains (TIGIT), T-cell immunoglobulin and mucin-3 (TIM-3), and lymphocyte-activation-gene-3 (LAG-3). Not only are activating and inhibitory receptors important for stimulating NK cell cytotoxicity but they also strictly control cytokine and chemokine secretions that further drive antitumor reactions (23). In addition to stimulation by activating receptors, NK cell activity can be enhanced by certain cytokines, notably IFN-γ, interleukin (IL)-2, IL-12, IL-15, IL-18, and IL-21 (24–29).

Two broad subsets of NK cells displaying different forms of anticancer activity are commonly recognized. CD56^bright^–CD16^negative^ precursor NK cells play an immune regulatory role *via* chemokine and cytokine secretions that attract and activate antitumor cells from both innate and adaptive arms of the immune system, comprising CD8^+^ T-cells, dendritic cells, and Th1 cells (30–32). Cytokines, including IFN-γ, TNF-α, and GM-CSF, work individually to recruit, activate, and stimulate the proliferation of antitumor immune cells and induce the presentation of MHC class II molecules on antigen-presenting cells (33). Later stages of NK cell maturation and activation are characterized by a CD56^+/dim^–CD16^bright^ phenotype, with higher cytotoxic capacity through lytic granule exocytosis and antibody-dependent cell cytotoxicity (23, 30, 34, 35). More recently, these classical categories have been put into question, where further activation of CD56^+/dim^–CD16^bright^ NK cells has demonstrated the functional reversibility of these cells to a predominantly IFN-γ-secreting role with lesser cytotoxicity, described as CD56^bright^–CD16^low/negative^ NK cells. Such a phenomenon has been coined “split anergy” (36, 37). Additional phenotypical analysis of tissue-resident NK cells and NK cells in peripheral blood and bone marrow has provided insight on a broad spectrum of NK cells (38). The differential role of these subsets in mediating a regulatory versus cytotoxic function against cancer continues to be investigated.

In cHL, the infiltration and activation of NK cells confers a favorable prognosis. Naranjo et al. found that a lower number of infiltrating activated CD56^dim^–CD16^bright^–CD57^+^ NK cells in cHL patients were associated with adverse prognostic factors, including the presence of B symptoms and advanced clinical stage (39). Nonetheless, NK cells remain largely decreased in cHL TME and fail to kill RS cells (40, 41).

## cHL Induces a Quantitative and Qualitative NK Cell Deficiency

Early studies of biopsies from cHL patients show a significant deficiency in NK cell numbers, with functional impairment in cytotoxicity. By looking at the *in situ* quantification of immune cells in cHL-affected lymphoid tissues, Gattringer et al. found NK cell density in cHL-affected tissues to be five times less compared to that of normal tissues and non-Hodgkin lymphoma (HL)-affected tissues, regardless of histological subtype (40). In addition, using the chromium release assay to measure cytotoxicity against the leukemic cell line K562, others have shown NK cells from spleens of cHL patients to be significantly less active than those of healthy donors (42). This impairment was amplified when cHL patients had B symptoms, suggesting a systemic response.

Concomitantly, a quantitative decrease in peripheral blood NK cells in cHL patients has also been observed, without correlation to adverse prognosis or advanced clinical stage (43). More importantly, peripheral blood NK cells in cHL patients are less cytotoxic, regardless of the stage or histological subtype (44–50).

Most recently, additional details on mechanisms behind the functional deficiency of NK cells in cHL patients have emerged. Reiners et al. observed feeble cytolysis of cHL-derived NK cells against the cHL cell line L428, in contrast to efficient killing by healthy donor NK cells (51). They found a significant reduction in NKG2D expression on untreated cHL-patient NK cells, without changes in other activating receptors or the markers, CD25 and CD69.

## cHL Mechanisms for NK Cell Inhibition

**Figure 1:**
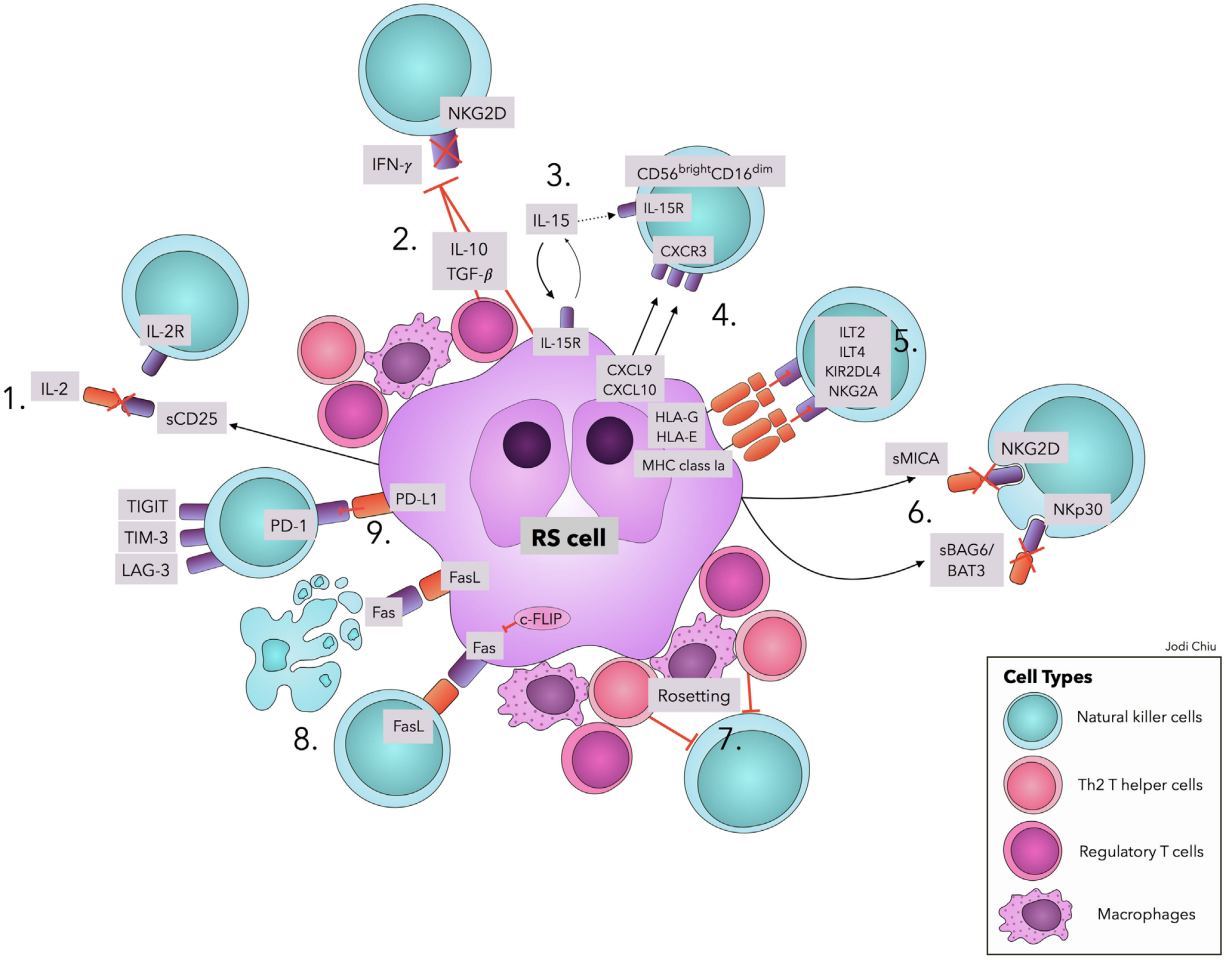
Natural killer cell evasion mechanisms in cHL. The accumulation of NK cell evasion mechanisms in cHL TME explains the persistent failure of NK cell infiltration and activity observed in patients. (1) sCD25 blocks IL-2 interaction with IL-2Rs on NK cells. (2) TGF-β and IL-10 repress IL-2 and IFN-γ production and downregulate NKG2D expression. (3) IL-15 is used by RS cells for proliferation and survival, diminishing available IL-15 in TME for NK cells. (4) Upregulation of CXCL9 and CXCL10 on RS cells attracts CXCR3-expressing CD56^bright^–CD16^dim^ NK cells, with lower efficacy in RS killing. (5) Interactions of MHC class I molecules, HLA-G, and HLA-E with corresponding inhibitory receptors suppress NK cell activity. (6) sMICA and sBAG6/BAT3 lead to NKG2D and NKp30 downregulation, respectively. (7) A physical barrier (“Rosetting”) consisting of Th2 T-helper cells, regulatory T cells, and macrophages shields RS cells from NK cells. (8) RS cells avoid Fas-mediated apoptosis by overexpressing c-FLIP. Expression of FasL on RS cells leads to apoptosis of Fas-expressing NK cells. (9) Interaction of PD-L1 with the cognate receptor PD-1 inhibits NK cell activation. Ligands in cHL for immune checkpoints TIGIT, TIM-3, and LAG-3 remain to be explored. Abbreviations: c-FLIP, cellular FLICE-inhibitory protein; cHL, classic Hodgkin Lymphoma; FasL, Fas ligand; IFN-γ, interferon-γ; IL, interleukin; IL-2R, IL-2 receptors; ILT, Immunoglobulin-like transcript; KIRs, Killer cell immunoglobulin-like receptor; LAG-3, lymphocyte-activation-gene-3; NK, natural killer; PD-1, Programmed cell death protein-1; PD-L1, Programmed death-ligand 1; RS, Reed–Sternberg; s, soluble; TGF-β, Transforming Growth Factor-β; TIGIT, T-cell immunoreceptor with Ig and ITIM domains; TIM-3, T-cell immunoglobulin and mucin-3.

Several factors contribute to the quantitative and functional deficiency of NK cells in cHL, including molecules and surface ligands produced and expressed by RS cells and the surrounding inflammatory milieu. We address those with evidence that directly and specifically promotes NK cell dysfunction in cHL as summarized in Table 1 and Figure 1.

**Table 1:** NK cell evasion mechanisms in cHL.

Mechanism	Source	Description
Soluble CD25	RS cells	Prevent interaction of IL-2 with IL-2Rs
IL-10	RS cells, Tregs, cells of TME	Repress IL-2 and IFN-γ production
TGF-β	RS cells, Tregs, cells of TME	Repress IL-2 and IFN-γ productionDownregulate activating receptors (NKG2D, NKp30) and corresponding ligands (MICA, ULBP2, ULBP4)
IL-15	RS cells	Competition of RS cells and NK cells
CXCL9, CXCL10	RS cells (mainly EBV^+^)	Attract CD56^bright^–CD16^dim^ NK cells
HLA-G and HLA-E	RS cells	Bind to inhibitory receptors on NK cells
Soluble MICA	RS cells	Endocytosis and degradation of NKG2D
BAG6/BAT3	RS cells	Endocytosis and degradation of NKp30
Rosetting	Macrophages, Tregs, Th2 T-helper cells	Physical shield of HRS cells from NK cells
c-FLIP	Overexpressed by RS cells	NK FasL-mediated apoptosis resistance
FasL	RS cells	Apoptosis of Fas-expressing NK cells
PD-L1	RS cells	Suppression of NK cell activation
MHC-I	RS cells (EBV^+^)	Bind to KIRs, inhibit NK cell activation

*c-FLIP, cellular FLICE-inhibitory protein; cHL, classic Hodgkin’s Lymphoma; EBV, Epstein–Barr Virus; FasL, Fas Ligand; IFN-γ, Interferon-gamma; IL, Interleukin; IL-2Rs, Interleukin-2 receptors; LAG-3, Lymphocyte-activation-gene-3; NK, Natural Killer cells; PD-1, Programmed Cell Death Protein-1; RS, Reed–Sternberg cells; TGF-β, Transforming Growth Factor-β; TIM-3, T-cell immunoglobulin and mucin-domain containing-3; TME, Tumor Microenvironment; Tregs, regulatory T-cells*.

### Cytokines and Chemokines: IL-2, IL-10, TGF-β, IL-15, CXCL9, and CXCL10

One significant mechanism of NK cell evasion that explains the persistent failure of NK cell lysis in cHL is the inhibition of IL-2, necessary for NK cell proliferation and activation (24). IL-2 is produced mainly by CD4^+^ T-cells, but also by activated CD8^+^ T-cells, dendritic cells, and NK cells themselves (52). However, IL-2 has been found to be largely absent from cHL TME (53–56). Moreover, cHL-patient-derived NK cells are unresponsive to exogenous IL-2 administration. cHL-derived NK cells and healthy donor NK cells fail to respond to IL-2 in the presence of cHL-patient serum, while observing expected enhancement effects in the absence of the serum (51). These findings can be attributed to the production of soluble IL-2Rα (sCD25) by RS cells, which bind IL-2 and prevent IL-2 interaction with T- and NK cells (57, 58). The observations are consistent with studies showing that higher levels of sCD25 in cHL are associated with poorer prognosis and advanced disease (59, 60).

In addition to IL-2, the immunosuppressive cytokines, IL-10 and TGF-β, are actively secreted by RS and TME cells (61). Both favor Treg recruitment and expansion (62) and reduce lymphocyte production of IFN-γ (63) involved in attracting NK cells (18). TGF-β can also diminish the expression of NKG2D ligands (MICA, ULBP2, and ULBP4) and downregulate activating receptors NKG2D and NKp30 (NCR) (64–66). TGF-β has been shown to directly mediate the transformation of NK cells into tumor-tolerant type 1 innate lymphoid cells in TME (67). IL-15 remains a surprising evasion mechanism in cHL due to its expected role in the differentiation and survival of NK cells (27). Ullrich et al. found that cHL cell lines upregulate IL-15 and corresponding receptors, demonstrating that RS cells utilize IL-15 for growth and apoptosis resistance in an autocrine fashion, thereby competing with NK cells (68).

CXCL9 and CXCL10 are ligands for CXCR3 and attract CXCR3-expressing NK cells (18, 69). Both chemokines are upregulated in cHL tissues and expressed at even higher levels in Epstein–Barr Virus (EBV)^+^ cHL (19, 70). However, because CXCR3 expression remains limited mostly to the less cytolytic NK cell subset (71), CXCL9 and CXCL10 favor the attraction of CD56^bright^–CD16^negative^ NK cells. TGF-β further increases CXCR3 expression, enabling an increased attraction of the CD56^bright^ subset toward CXCL9 and CXCL10 (72). Although the failure of CD56^bright^–CD16^negative^ NK cells to kill RS cells is not entirely understood, recent studies on the genetic and molecular characteristics of RS have provided evidence of intrinsic resistance to cytokine-mediated apoptosis. Using flow sorting and exome sequencing of primary RS cells, Reichel et al. demonstrated that tumor necrosis factor alpha-induced protein 3, normally responsible for inhibiting NFκB survival pathway and mediating TNF-α-induced apoptosis, is the second most common mutation (73).

### HLA-G and HLA-E

Reed–Sternberg cells have long been known to lack the expression of MHC class I proteins (41, 73), putting them at risk of NK cell lysis due to loss of “self” ligands for inhibitory KIRs. Thus, RS cells evade NK cell cytotoxicity by upregulating HLA-G and HLA-E. HLA-G is a ligand for the inhibitory receptors, immunoglobulin-like transcript (ILT) 2, ILT4, and killer immunoglobulin-like receptor KIR2DL4 (p49) (74, 75), while HLA-E interacts with CD94/NKG2A (76). Notably, HLA-G has been found to stain positive in more than 50% of cHL-lymph node specimens (41), while HLA-E tests positive in 70 and 62.5% of RS cells and TME lymphocytes, respectively (76). HLA-E also correlates with advanced clinical stage.

### Soluble Ligands

Two NK cell-activating signals often interrupted in cHL are surface receptors NKp30 and NKG2D (51). MICA, ligand for NKG2D, and BAG6/BAT3, ligand for NKp30, are only expressed on cell surfaces upon stress or damage to alert and activate immune cells (77, 78). RS cells overcome this threat by releasing both soluble ligands, predominantly using protein disulfide isomerase ERp5, and disintegrins and metalloproteinases ADAM10 and ADAM17 (79). In fact, MICA and BAG6/BAT3 can be significantly elevated in the serum of untreated cHL patients (51). Not only are these soluble ligands ineffective in activating NKG2D and NKp30 but they also result in receptor endocytosis and degradation (80).

### Physical Barriers

Another evasion mechanism is the formation of a protective barrier around RS cells using Tregs, Th2 cells, and macrophages, termed “rosetting” (81). This rosetting exhibits physical binding characteristics that are not easily destroyed. Using immunostaining, Hartmann et al. detected CD4^+^ T-cell and CD163 macrophage rosetting encompassing RS cells to be present in 14 of 15 cHL cases examined (82). The maintenance of a close contact between RS cells with CD4^+^ T-cells and CD163 macrophages suggests their role in physically shielding malignant cells from NK cell attack.

### Fas/Fas ligand (FasL)

Although RS cells largely express extrinsic death receptor Fas, they avoid extrinsic apoptosis induced by FasL-expressing NK cells by overexpressing cellular FLICE-inhibitory protein (83). In addition, RS cells overexpress FasL in 87% of cases, making them capable of inducing apoptosis on Fas-expressing NK cells (84, 85).

### Immune Checkpoints

A near universal expression of immune checkpoints has been demonstrated among TME and RS cells (86, 87). A group of genetic alterations in the loci of PD-1 ligands, PD-L1 and PD-L2, were found in 97% of cHL cases (88). Of significance, the amplification of locus 9p24.1 correlated with advanced clinical stages and worse progression-free survival. PD-1 and PD-L1 expression in patient biopsies are also of prognostic significance: a high expression of PD-1 and PD-L1 correlated with a lower event-free survival, while a high expression of PD-L1 correlated with a lower overall survival (89). NK cells and CD8^+^ T-cells in cHL TME can, therefore, be directly inhibited upon the expression of PD-1. The development of the anti-PD-1 monoclonal antibodies, Nivolumab and Pembrolizumab, was aimed to prevent such inhibition. A majority of relapsed and refractory cHL patients showed responses to treatments (65 and 87%, respectively) (89). The role of NK cell-immune checkpoints, LAG-3, TIM-3, and TIGIT, remains unknown in cHL.

### EBV^+^ cHL

Classic Hodgkin lymphoma cells are infected with EBV in approximately 40% of cases (90). EBV^+^ cHL TME is characterized by the predominance of CD8^+^ T-, Th1, and NK cells, significantly contrasting the EBV-negative phenotype. Despite the presence of additional cytotoxic cells in EBV^+^ cHL, this has minimal influence on prognosis (91, 92), suggesting effective evasion. EBV induces an upregulation of MHC class I molecules in approximately 70% of cases, encouraging self-tolerance in NK cells through inhibitory KIRs (41). In addition, EBV^+^ Tregs secrete twofold more IL-10, increasing immunosuppression in TME (92).

## The Reversibility of Evasion Mechanisms

Despite numerous evasion mechanisms, observations have been made on their reversibility after achieving remission with chemo and/or radiotherapy (47, 93). NK cell cytotoxicity is significantly diminished at cHL diagnosis, independent of clinical stage. This functional deficiency of NK cells normalized 6 weeks after completion of the treatment protocol, contrasting with the long-lasting cellular immune suppression of T-cells. Moreover, the failure to respond or the evidence of early relapse does not appear to improve NK cell function, suggesting that NK cell activity is a biomarker of clinical response and prognosis.

Immune reconstitution after autologous hematopoietic cell transplantation (AHCT) for relapsed or refractory cHL also demonstrates the reversibility of immune suppression. Patients with early recovery (day 15 post AHCT) of the absolute lymphocyte count defined as greater than 500 × 10^9^/L in one study, and greater than 667 × 10^9^/L in another, had a significantly higher progression-free (in both studies) and overall survival (one study) (94, 95). NK cell counts can rapidly return to normal as early as 2 weeks post AHCT (96), while T- and B-cells remain deficient for months to years. This not only implies early reversibility of NK cell evasion mechanisms after treatment but also highlights the importance of NK cells in preventing relapse early after transplant.

## Potential for NK-Targeted Immunotherapies in cHL

One approach to reactivate silenced NK cells in cHL is to employ the currently clinically available monoclonal antibodies, Nivolumab and Pembrolizumab, to block immune checkpoint PD-1 on activated CD4^+^ and CD8^+^ T-cells, and NK cells (97). Several other molecules targeting NK cell reactivation are under investigation. Heat shock protein-90 inhibitor, BIIB021, is effective against cHL in preclinical studies *in vitro* and *in vivo* (97). It acts by directly blocking the NFκB pathway and potentiating NK cell-directed lysis through downregulating MHC class I molecules and upregulating NKG2D ligands MICA, MICB, and ULBP2. A different approach to NK cell immunotherapy has been the study of ADAM10/17 inhibitors. Although still in preclinical testing, ADAM10/17 inhibitors have shown high specificity in their activity and with high affinity (IC_50_ 40 nM for ADAM10) (98, 99). More recently, the tetravalent bispecific CD30/CD16A tandem antibody, AFM13, has been developed to boost autologous NK cells against RS cells (51, 100). A phase I clinical trial of relapsed and refractory cHL showed that the drug was safe and tolerable, with minimal toxicities (101). Patients receiving AFM13 showed an increase in the NK cell activation marker, CD69, after each dose with preliminary evidence of efficacy. A phase II trial with AFM13 is currently underway (GHSG-AFM13 and NCT02321592).

Numerous clinical trials have employed allogeneic NK cells derived from healthy donors with variable outcomes (102). An alternative approach is to use an allogeneic permanent, malignant NK cell line, NK-92, derived from a patient with an NK lymphoma. The parental NK-92 line lacks CD16 expression and hence cannot engage in antibody-dependent cytotoxicity, and its cytolytic activity, lower than for primary NK cells, relies heavily on the activation of the activating receptors NKp30, NKp46, and NKG2D, and the absence of most inhibitory KIRs (103). In addition, given the malignant origin of NK-92, irradiation prior to infusion is required, reducing its cytotoxicity further, including IFN-γ secretion, compared with primary NK cells (28, 104, 105). NK-92 cells nonetheless can kill a variety of cancer cell types (104), and the line is a potentially universal, off-the-shelf source of readily expanded NK cells with uniform cytotoxicity and a high safety profile (106). We recently reported a phase I trial of NK-92 in patients with hematological malignancies relapsing after hematopoietic cell transplantation and found the treatment to be well tolerated and documented several responses, including a patient with refractory cHL who has remained in unmaintained remission for 11 years after NK-92 infusion (107) by an uncertain mechanism.

## Future Directions

Not only do 15% of cHL patients fail to achieve long-term remission (1, 2), but accumulating evidence indicates that significant long-term toxicities result from aggressive management with chemotherapy and radiotherapy, including an increased risk of mortality from solid tumors and cardiovascular disease, as well as increased risks in the development of second cancer and diabetes mellitus (108–112). Consequently, studies that explore the immunotherapy of NK cell reactivation to improve long-term disease-free survival and promote harm reduction warrant further investigation.

## Conclusion

Classic Hodgkin lymphoma is characterized by a potent immunosuppressive TME that inhibits NK cells. It is worth noting that the quantitative and qualitative NK cell deficiencies exhibited by patients with cHL are reversible. Strategies to reactivate NK cell function or block the evasive mechanisms displayed by the TME need further investigation and are likely to identify new immunotherapeutic targets.

## Author Contributions

JC and DE wrote and revised the final manuscript; JC provided the figure; and AK contributed to manuscript editing and final revision.

## Conflict of Interest Statement

The authors declare that the research was conducted in the absence of any commercial or financial relationships that could be construed as a potential conflict of interest.

## References

[1] Society AC Cancer Facts & Figures. Atlanta (2017).

[2] Ferlay J GLOBOCAN 2012 v1.0, Cancer Incidence and Mortality Worldwide. IARC CancerBase. No. 11. Lyon (2013). Available from: http://globocan.iarc.fr2013/

[3] Yung L Linch D Hodgkin’s lymphoma. Lancet (2003) 361(9361):943–51. 10.1016/S0140-6736(03)12777-8 12648984

[4] Gobbi PG Ferreri AJ Ponzoni M Levis A. Hodgkin lymphoma. Crit Rev Oncol Hematol (2013) 85(2):216–37. 10.1016/j.critrevonc.2012.07.002 22867814

[5] Ansell SM Lesokhin AM Borrello I Halwani A Scott EC Gutierrez M PD-1 blockade with Nivolumab in relapsed or refractory Hodgkin’s lymphoma. N Engl J Med (2015) 372(4):311–9. 10.1056/NEJMoa1411087 25482239 PMC4348009

[6] Younes A Santoro A Shipp M Zinzani PL Timmerman JM Ansell S Nivolumab for classical Hodgkin’s lymphoma after failure of both autologous stem-cell transplantation and brentuximab vedotin: a multicentre, multicohort, single-arm phase 2 trial. Lancet Oncol (2016) 17(9):1283–94. 10.1016/S1470-2045(16)30167-X 27451390 PMC5541855

[7] Chen R Zinzani PL Fanale MA Armand P Johnson NA Brice P Phase II study of the efficacy and safety of Pembrolizumab for relapsed/refractory classic Hodgkin lymphoma. J Clin Oncol (2017) 35(19):2125–32. 10.1200/JCO.2016.72.1316 28441111 PMC5791843

[8] Topalian SL Drake CG Pardoll DM. Immune checkpoint blockade: a common denominator approach to cancer therapy. Cancer Cell (2015) 27(4):450–61. 10.1016/j.ccell.2015.03.001 25858804 PMC4400238

[9] Morvan MG Lanier LL. NK cells and cancer: you can teach innate cells new tricks. Nat Rev Cancer (2016) 16(1):7–19. 10.1038/nrc.2015.5 26694935

[10] Iannello A Thompson TW Ardolino M Marcus A Raulet DH. Immunosurveillance and immunotherapy of tumors by innate immune cells. Curr Opin Immunol (2016) 38:52–8. 10.1016/j.coi.2015.11.001 26686774 PMC4715905

[11] Liu Y Sattarzadeh A Diepstra A Visser L van den Berg A. The microenvironment in classical Hodgkin lymphoma: an actively shaped and essential tumor component. Semin Cancer Biol (2014) 24:15–22. 10.1016/j.semcancer.2013.07.002 23867303

[12] Schreck S Friebel D Buettner M Distel L Grabenbauer G Young LS Prognostic impact of tumour-infiltrating Th2 and regulatory T cells in classical Hodgkin lymphoma. Hematol Oncol (2009) 27(1):31–9. 10.1002/hon.878 18924115

[13] Cattaruzza L Gloghini A Olivo K Di Francia R Lorenzon D De Filippi R Functional coexpression of interleukin (IL)-7 and its receptor (IL-7R) on Hodgkin and Reed–Sternberg cells: involvement of IL-7 in tumor cell growth and microenvironmental interactions of Hodgkin’s lymphoma. Int J Cancer (2009) 125(5):1092–101. 10.1002/ijc.24389 19391137

[14] Chemnitz JM Driesen J Classen S Riley JL Debey S Beyer M Prostaglandin E2 impairs CD4^+^ T cell activation by inhibition of lck: implications in Hodgkin’s lymphoma. Cancer Res (2006) 66(2):1114–22. 10.1158/0008-5472.CAN-05-3252 16424048

[15] Chemnitz JM Eggle D Driesen J Classen S Riley JL Debey-Pascher S RNA fingerprints provide direct evidence for the inhibitory role of TGFbeta and PD-1 on CD4^+^ T cells in Hodgkin lymphoma. Blood (2007) 110(9):3226–33. 10.1182/blood-2006-12-064360 17644739

[16] Gandhi MK Moll G Smith C Dua U Lambley E Ramuz O Galectin-1 mediated suppression of Epstein–Barr virus specific T-cell immunity in classic Hodgkin lymphoma. Blood (2007) 110(4):1326–9. 10.1182/blood-2007-01-066100 17438085 PMC1939905

[17] Juszczynski P Ouyang J Monti S Rodig SJ Takeyama K Abramson J The AP1-dependent secretion of galectin-1 by Reed–Sternberg cells fosters immune privilege in classical Hodgkin lymphoma. Proc Natl Acad Sci U S A (2007) 104(32):13134–9. 10.1073/pnas.0706017104 17670934 PMC1936978

[18] Wendel M Galani IE Suri-Payer E Cerwenka A. Natural killer cell accumulation in tumors is dependent on IFN-gamma and CXCR3 ligands. Cancer Res (2008) 68(20):8437–45. 10.1158/0008-5472.CAN-08-1440 18922917

[19] Maggio EM Van Den Berg A Visser L Diepstra A Kluiver J Emmens R Common and differential chemokine expression patterns in RS cells of NLP, EBV positive and negative classical Hodgkin lymphomas. Int J Cancer (2002) 99(5):665–72. 10.1002/ijc.10399 12115499

[20] Ohshima K Karube K Hamasaki M Tutiya T Yamaguchi T Suefuji H Differential chemokine, chemokine receptor and cytokine expression in Epstein–Barr virus-associated lymphoproliferative diseases. Leuk Lymphoma (2003) 44(8):1367–78. 10.1080/1042819031000082984 12952231

[21] Staege MS. A multi-component model of Hodgkin’s lymphoma. PLoS One (2015) 10(4):e0124614. 10.1371/journal.pone.0124614 25915037 PMC4411114

[22] Martinet L Smyth MJ. Balancing natural killer cell activation through paired receptors. Nat Rev Immunol (2015) 15(4):243–54. 10.1038/nri3799 25743219

[23] Fauriat C Long EO Ljunggren HG Bryceson YT. Regulation of human NK-cell cytokine and chemokine production by target cell recognition. Blood (2010) 115(11):2167–76. 10.1182/blood-2009-08-238469 19965656 PMC2844017

[24] Yu TK Caudell EG Smid C Grimm EA. IL-2 activation of NK cells: involvement of MKK1/2/ERK but not p38 kinase pathway. J Immunol (2000) 164(12):6244–51. 10.4049/jimmunol.164.12.6244 10843677

[25] Ferlazzo G Pack M Thomas D Paludan C Schmid D Strowig T Distinct roles of IL-12 and IL-15 in human natural killer cell activation by dendritic cells from secondary lymphoid organs. Proc Natl Acad Sci U S A (2004) 101(47):16606–11. 10.1073/pnas.0407522101 15536127 PMC534504

[26] Heidemann SC Chavez V Landers CJ Kucharzik T Prehn JL Targan SR. TL1A selectively enhances IL-12/IL-18-induced NK cell cytotoxicity against NK-resistant tumor targets. J Clin Immunol (2010) 30(4):531–8. 10.1007/s10875-010-9382-9 20349123 PMC2900590

[27] Marcais A Cherfils-Vicini J Viant C Degouve S Viel S Fenis A The metabolic checkpoint kinase mTOR is essential for IL-15 signaling during the development and activation of NK cells. Nat Immunol (2014) 15(8):749–57. 10.1038/ni.2936 24973821 PMC4110708

[28] Strengell M Matikainen S Siren J Lehtonen A Foster D Julkunen I IL-21 in synergy with IL-15 or IL-18 enhances IFN- production in human NK and T cells. J Immunol (2003) 170(11):5464–9. 10.4049/jimmunol.170.11.5464 12759422

[29] McMichael EL Jaime-Ramirez A Guenterberg K Luedke E Atwal L Carson WE Interleukin-21 activates natural killer cell activity against cetuximab-coated pancreatic tumor cells. J Immunother Cancer (2015) 3(Suppl 2):233 10.1186/2051-1426-3-S2-P233

[30] Cooper MA Fehniger TA Turner SC Chen KS Ghaheri BA Ghayur T Human natural killer cells: a unique innate immunoregulatory role for the CD56(bright) subset. Blood (2001) 97(10):3146–51. 10.1182/blood.V97.10.3146 11342442

[31] Martin-Fontecha A Thomsen LL Brett S Gerard C Lipp M Lanzavecchia A Induced recruitment of NK cells to lymph nodes provides IFN-gamma for T(H)1 priming. Nat Immunol (2004) 5(12):1260–5. 10.1038/ni1138 15531883

[32] Michel T Poli A Cuapio A Briquemont B Iserentant G Ollert M Human CD56^bright^ NK cells: an update. J Immunol (2016) 196(7):2923–31. 10.4049/jimmunol.1502570 26994304

[33] Bajenoff M Breart B Huang AY Qi H Cazareth J Braud VM Natural killer cell behavior in lymph nodes revealed by static and real-time imaging. J Exp Med (2006) 203(3):619–31. 10.1084/jem.20051474 16505138 PMC2118232

[34] Beziat V Duffy D Quoc SN Le Garff-Tavernier M Decocq J Combadiere B CD56^bright^CD16^+^ NK cells: a functional intermediate stage of NK cell differentiation. J Immunol (2011) 186(12):6753–61. 10.4049/jimmunol.1100330 21555534

[35] Lopez-Verges S Milush JM Pandey S York VA Arakawa-Hoyt J Pircher H CD57 defines a functionally distinct population of mature NK cells in the human CD56^dim^CD16^+^ NK-cell subset. Blood (2010) 116(19):3865–74. 10.1182/blood-2010-04-282301 20733159 PMC2981540

[36] Tseng HC Arasteh A Kaur K Kozlowska A Topchyan P Jewett A Differential cytotoxicity but augmented IFN-gamma secretion by NK cells after interaction with monocytes from humans, and those from wild type and myeloid-specific COX-2 knockout mice. Front Immunol (2015) 6:259 10.3389/fimmu.2015.00259 26106386 PMC4460808

[37] Jewett A Man YG Cacalano N Kos J Tseng HC. Natural killer cells as effectors of selection and differentiation of stem cells: role in resolution of inflammation. J Immunotoxicol (2014) 11(4):297–307. 10.3109/1547691X.2013.877104 24575813

[38] Freud AG Mundy-Bosse BL Yu J Caligiuri MA. The broad spectrum of human natural killer cell diversity. Immunity (2017) 47(5):820–33. 10.1016/j.immuni.2017.10.008 29166586 PMC5728700

[39] Alvaro-Naranjo T Lejeune M Salvado-Usach MT Bosch-Princep R Reverter-Branchat G Jaen-Martinez J Tumor-infiltrating cells as a prognostic factor in Hodgkin’s lymphoma: a quantitative tissue microarray study in a large retrospective cohort of 267 patients. Leuk Lymphoma (2005) 46(11):1581–91. 10.1080/10428190500220654 16236613

[40] Gattringer G Greil R Radaszkiewicz T Huber H. *In situ* quantification of T-cell subsets, NK-like cells and macrophages in Hodgkin’s disease: quantity and quality of infiltration density depends on histopathological subtypes. Blut (1986) 53(1):49–58. 10.1007/BF00320582 3487362

[41] Diepstra A Poppema S Boot M Visser L Nolte IM Niens M HLA-G protein expression as a potential immune escape mechanism in classical Hodgkin’s lymphoma. Tissue Antigens (2008) 71(3):219–26. 10.1111/j.1399-0039.2008.01005.x 18257895

[42] Al Sam S Jones DB Payne SV Wright DH Natural killer (NK) activity in the spleen of patients with Hodgkin’s disease and controls. Br J Cancer (1982) 46(5):806–10. 10.1038/bjc.1982.274 7171458 PMC2011174

[43] Ayoub JP Palmer JL Huh Y Cabanillas F Younes A. Therapeutic and prognostic implications of peripheral blood lymphopenia in patients with Hodgkin’s disease. Leuk Lymphoma (1999) 34(5–6):519–27. 10.3109/10428199909058479 10492075

[44] Tursz T Dokhelar MC Lipinski M Amiel JL. Low natural killer cell activity in patients with malignant lymphoma. Cancer (1982) 50(11):2333–5. 10.1002/1097-0142(19821201)50:11<2333::AID-CNCR2820501119>3.0.CO;2-W 6958348

[45] Hawrylowicz CM Rees RC Hancock BW Potter CW. Depressed spontaneous natural killing and interferon augmentation in patients with malignant lymphoma. Eur J Cancer Clin Oncol (1982) 18(11):1081–8. 10.1016/0277-5379(82)90087-6 6891651

[46] Healy F Rees RC Hancock BW. An assessment of natural cell-mediated cytotoxicity in patients with malignant lymphoma. Eur J Cancer Clin Oncol (1985) 21(7):775–83. 10.1016/0277-5379(85)90215-9 2412830

[47] Frydecka I. Natural killer cell activity during the course of disease in patients with Hodgkin’s disease. Cancer (1985) 56(12):2799–803. 10.1002/1097-0142(19851215)56:12<2799::AID-CNCR2820561215>3.0.CO;2-W 4052954

[48] Komiyama A Kawai H Yamada S Kato M Yanagisawa M Miyagawa Y A killing defect of natural killer cells with the absence of natural killer cytotoxic factors in a child with Hodgkin’s disease. Blood (1987) 69(6):1686–90. 3580573

[49] Rajaram N Tatake RJ Advani SH Gangal SG. Natural killer and lymphokine activated killer cell functions in Hodgkin’s disease. Br J Cancer (1990) 62(2):205–8. 10.1038/bjc.1990.261 2386735 PMC1971824

[50] Konjevic G Jurisic V Banicevic B Spuzic I. The difference in NK-cell activity between patients with non-Hodgkin’s lymphomas and Hodgkin’s disease. Br J Haematol (1999) 104(1):144–51. 10.1046/j.1365-2141.1999.01129.x 10027727

[51] Reiners KS Kessler J Sauer M Rothe A Hansen HP Reusch U Rescue of impaired NK cell activity in Hodgkin lymphoma with bispecific antibodies *in vitro* and in patients. Mol Ther (2013) 21(4):895–903. 10.1038/mt.2013.14 23459515 PMC3616527

[52] Rosenberg SA. IL-2: the first effective immunotherapy for human cancer. J Immunol (2014) 192(12):5451–8. 10.4049/jimmunol.1490019 24907378 PMC6293462

[53] Damle RN Advani SH Gangal SG. Impairment in proliferation, lymphokine production and frequency distribution of mitogen-responsive and interleukin-2-producing cells in Hodgkin’s disease. Cancer Immunol Immunother (1991) 34(3):205–10. 10.1007/BF01742314 1756538 PMC11037982

[54] Ford RJ Tsao J Kouttab NM Sahasrabuddhe CG Mehta SR. Association of an interleukin abnormality with the T cell defect in Hodgkin’s disease. Blood (1984) 64(2):386–92. 6611180

[55] Liberati AM Ballatori E Fizzotti M Schippa M Proietti MG Di Marzio R Immunologic profile in patients with Hodgkin’s disease in complete remission. Cancer (1987) 59(11):1906–13. 10.1002/1097-0142(19870601)59:11<1906::AID-CNCR2820591111>3.0.CO;2-A 3105863

[56] Kosmaczewska A Frydecka I Bocko D Ciszak L Teodorowska R. Correlation of blood lymphocyte CTLA-4 (CD152) induction in Hodgkin’s disease with proliferative activity, interleukin 2 and interferon-gamma production. Br J Haematol (2002) 118(1):202–9. 10.1046/j.1365-2141.2002.03572.x 12100149

[57] Damle RN Advani SH Gangal SG. Analysis of regulation of T-cell responses by soluble inhibitory factors from the sera of patients with Hodgkin’s disease. Int J Cancer (1992) 50(2):192–6. 10.1002/ijc.2910500206 1730512

[58] Gooding R Riches P Dadian G Moore J Gore M. Increased soluble interleukin-2 receptor concentration in plasma predicts a decreased cellular response to IL-2. Br J Cancer (1995) 72(2):452–5. 10.1038/bjc.1995.354 7640231 PMC2033994

[59] Ambrosetti A Nadali G Vinante F Carlini S Veneri D Todeschini G Serum levels of soluble interleukin-2 receptor in Hodgkin disease. Relationship with clinical stage, tumor burden, and treatment outcome. Cancer (1993) 72(1):201–6. 10.1002/1097-0142(19930701)72:1<201::AID-CNCR2820720136>3.0.CO;2-V 8508408

[60] Viviani S Camerini E Bonfante V Santoro A Balzarotti M Fornier M Soluble interleukin-2 receptors (sIL-2R) in Hodgkin’s disease: outcome and clinical implications. Br J Cancer (1998) 77(6):992–7. 10.1038/bjc.1998.163 9528846 PMC2150083

[61] Marshall NA Christie LE Munro LR Culligan DJ Johnston PW Barker RN Immunosuppressive regulatory T cells are abundant in the reactive lymphocytes of Hodgkin lymphoma. Blood (2004) 103(5):1755–62. 10.1182/blood-2003-07-2594 14604957

[62] Hsu P Santner-Nanan B Hu M Skarratt K Lee CH Stormon M IL-10 potentiates differentiation of human induced regulatory T cells *via* STAT3 and Foxo1. J Immunol (2015) 195(8):3665–74. 10.4049/jimmunol.1402898 26363058

[63] Schroder M Meisel C Buhl K Profanter N Sievert N Volk HD Different modes of IL-10 and TGF- to inhibit cytokine-dependent IFN- production: consequences for reversal of lipopolysaccharide desensitization. J Immunol (2003) 170(10):5260–7. 10.4049/jimmunol.170.10.5260 12734375

[64] Eisele G Wischhusen J Mittelbronn M Meyermann R Waldhauer I Steinle A TGF-beta and metalloproteinases differentially suppress NKG2D ligand surface expression on malignant glioma cells. Brain (2006) 129(Pt 9):2416–25. 10.1093/brain/awl205 16891318

[65] Castriconi R Dondero A Bellora F Moretta L Castellano A Locatelli F Neuroblastoma-derived TGF-beta1 modulates the chemokine receptor repertoire of human resting NK cells. J Immunol (2013) 190(10):5321–8. 10.4049/jimmunol.1202693 23576682

[66] Lee JC Lee KM Kim DW Heo DS Elevated TGF- 1 secretion and down-modulation of NKG2D underlies impaired NK cytotoxicity in cancer patients. J Immunol (2004) 172(12):7335–40. 10.4049/jimmunol.172.12.7335 15187109

[67] Gao Y Souza-Fonseca-Guimaraes F Bald T Ng SS Young A Ngiow SF Tumor immunoevasion by the conversion of effector NK cells into type 1 innate lymphoid cells. Nat Immunol (2017) 18(9):1004–15. 10.1038/ni.3800 28759001

[68] Ullrich K Blumenthal-Barby F Lamprecht B Kochert K Lenze D Hummel M The IL-15 cytokine system provides growth and survival signals in Hodgkin lymphoma and enhances the inflammatory phenotype of HRS cells. Leukemia (2015) 29(5):1213–8. 10.1038/leu.2014.345 25486870

[69] Teichmann M Meyer B Beck A Niedobitek G. Expression of the interferon-inducible chemokine IP-10 (CXCL10), a chemokine with proposed anti-neoplastic functions, in Hodgkin lymphoma and nasopharyngeal carcinoma. J Pathol (2005) 206(1):68–75. 10.1002/path.1745 15751051

[70] Teruya-Feldstein J Tosato G Jaffe ES. The role of chemokines in Hodgkin’s disease. Leuk Lymphoma (2000) 38(3–4):363–71. 10.3109/10428190009087027 10830743

[71] Campbell JJ Qin S Unutmaz D Soler D Murphy KE Hodge MR Unique subpopulations of CD56^+^ NK and NK-T peripheral blood lymphocytes identified by chemokine receptor expression repertoire. J Immunol (2001) 166(11):6477–82. 10.4049/jimmunol.166.11.6477 11359797

[72] Wu X Jin LP Yuan MM Zhu Y Wang MY Li DJ. Human first-trimester trophoblast cells recruit CD56^bright^CD16^−^ NK cells into decidua by way of expressing and secreting of CXCL12/stromal cell-derived factor 1. J Immunol (2005) 175(1):61–8. 10.4049/jimmunol.175.1.61 15972632

[73] Reichel J Chadburn A Rubinstein PG Giulino-Roth L Tam W Liu Y Flow sorting and exome sequencing reveal the oncogenome of primary Hodgkin and Reed–Sternberg cells. Blood (2015) 125(7):1061–72. 10.1182/blood-2014-11-610436 25488972

[74] Cantoni C Falco M Pessino A Moretta A Moretta L Biassoni R. P49, a putative HLA-G1 specific inhibitory NK receptor belonging to the immunoglobulin superfamily. J Reprod Immunol (1999) 43(2):157–65. 10.1016/S0165-0378(99)00031-5 10479051

[75] Shiroishi M Tsumoto K Amano K Shirakihara Y Colonna M Braud VM Human inhibitory receptors Ig-like transcript 2 (ILT2) and ILT4 compete with CD8 for MHC class I binding and bind preferentially to HLA-G. Proc Natl Acad Sci U S A (2003) 100(15):8856–61. 10.1073/pnas.1431057100 12853576 PMC166403

[76] Kren L Fabian P Slaby O Janikova A Soucek O Sterba J Multifunctional immune-modulatory protein HLA-E identified in classical Hodgkin lymphoma: possible implications. Pathol Res Pract (2012) 208(1):45–9. 10.1016/j.prp.2011.11.004 22177730

[77] Groh V Rhinehart R Randolph-Habecker J Topp MS Riddell SR Spies T. Costimulation of CD8alphabeta T cells by NKG2D *via* engagement by MIC induced on virus-infected cells. Nat Immunol (2001) 2(3):255–60. 10.1038/85321 11224526

[78] Pogge von Strandmann E Simhadri VR von Tresckow B Sasse S Reiners KS Hansen HP Human leukocyte antigen-B-associated transcript 3 is released from tumor cells and engages the NKp30 receptor on natural killer cells. Immunity (2007) 27(6):965–74. 10.1016/j.immuni.2007.10.010 18055229

[79] Zocchi MR Catellani S Canevali P Tavella S Garuti A Villaggio B High ERp5/ADAM10 expression in lymph node microenvironment and impaired NKG2D ligands recognition in Hodgkin lymphomas. Blood (2012) 119(6):1479–89. 10.1182/blood-2011-07-370841 22167753

[80] Binici J Hartmann J Herrmann J Schreiber C Beyer S Guler G A soluble fragment of the tumor antigen BCL2-associated athanogene 6 (BAG-6) is essential and sufficient for inhibition of NKp30 receptor-dependent cytotoxicity of natural killer cells. J Biol Chem (2013) 288(48):34295–303. 10.1074/jbc.M113.483602 24133212 PMC3843045

[81] Morris CS Stuart AE. Reed–Sternberg/lymphocyte rosette: lymphocyte subpopulations as defined by monoclonal antibodies. J Clin Pathol (1984) 37(7):767–71. 10.1136/jcp.37.7.767 6378976 PMC498806

[82] Hartmann S Jakobus C Rengstl B Doring C Newrzela S Brodt HR Spindle-shaped CD163^+^ rosetting macrophages replace CD4^+^ T-cells in HIV-related classical Hodgkin lymphoma. Mod Pathol (2013) 26(5):648–57. 10.1038/modpathol.2012.217 23307058

[83] Mathas S Lietz A Anagnostopoulos I Hummel F Wiesner B Janz M c-FLIP mediates resistance of Hodgkin/Reed–Sternberg cells to death receptor-induced apoptosis. J Exp Med (2004) 199(8):1041–52. 10.1084/jem.20031080 15078899 PMC2211891

[84] Verbeke CS Wenthe U Grobholz R Zentgraf H. Fas ligand expression in Hodgkin lymphoma. Am J Surg Pathol (2001) 25(3):388–94. 10.1097/00000478-200103000-00014 11224610

[85] Medvedev AE Johnsen AC Haux J Steinkjer B Egeberg K Lynch DH Regulation of Fas and Fas-ligand expression in NK cells by cytokines and the involvement of Fas-ligand in NK/LAK cell-mediated cytotoxicity. Cytokine (1997) 9(6):394–404. 10.1006/cyto.1996.0181 9199873

[86] Gandhi MK Lambley E Duraiswamy J Dua U Smith C Elliott S Expression of LAG-3 by tumor-infiltrating lymphocytes is coincident with the suppression of latent membrane antigen-specific CD8^+^ T-cell function in Hodgkin lymphoma patients. Blood (2006) 108(7):2280–9. 10.1182/blood-2006-04-015164 16757686

[87] Yamamoto R Nishikori M Kitawaki T Sakai T Hishizawa M Tashima M PD-1-PD-1 ligand interaction contributes to immunosuppressive microenvironment of Hodgkin lymphoma. Blood (2008) 111(6):3220–4. 10.1182/blood-2007-05-085159 18203952

[88] Roemer MG Advani RH Ligon AH Natkunam Y Redd RA Homer H PD-L1 and PD-L2 genetic alterations define classical Hodgkin lymphoma and predict outcome. J Clin Oncol (2016) 34(23):2690–7. 10.1200/JCO.2016.66.4482 27069084 PMC5019753

[89] Hollander P Kamper P Smedby KE Enblad G Ludvigsen M Mortensen J High proportions of PD-1^+^ and PD-L1^+^ leukocytes in classical Hodgkin lymphoma microenvironment are associated with inferior outcome. Blood Adv (2017) 1(18):1427–39. 10.1182/bloodadvances.2017006346 29296784 PMC5727849

[90] Glaser SL Lin RJ Stewart SL Ambinder RF Jarrett RF Brousset P Epstein–Barr virus-associated Hodgkin’s disease: epidemiologic characteristics in international data. Int J Cancer (1997) 70(4):375–82. 10.1002/(SICI)1097-0215(19970207)70:4<375::AID-IJC1>3.0.CO;2-T 9033642

[91] Keegan TH Glaser SL Clarke CA Gulley ML Craig FE Digiuseppe JA Epstein–Barr virus as a marker of survival after Hodgkin’s lymphoma: a population-based study. J Clin Oncol (2005) 23(30):7604–13. 10.1200/JCO.2005.02.6310 16186595

[92] Herling M Rassidakis GZ Medeiros LJ Vassilakopoulos TP Kliche KO Nadali G Expression of Epstein–Barr virus latent membrane protein-1 in Hodgkin and Reed–Sternberg cells of classical Hodgkin’s lymphoma: associations with presenting features, serum interleukin 10 levels, and clinical outcome. Clin Cancer Res (2003) 9(6):2114–20. 12796376

[93] Douer D Shaked N Ramot B. Normal natural killer cell activity in Hodgkin’s disease patients in remission. Clin Exp Immunol (1987) 69(3):660–7. 3665188 PMC1542390

[94] Porrata LF Inwards DJ Micallef IN Ansell SM Geyer SM Markovic SN. Early lymphocyte recovery post-autologous haematopoietic stem cell transplantation is associated with better survival in Hodgkin’s disease. Br J Haematol (2002) 117(3):629–33. 10.1046/j.1365-2141.2002.03478.x 12028034

[95] Gordan LN Sugrue MW Lynch JW Williams KD Khan SA Moreb JS. Correlation of early lymphocyte recovery and progression-free survival after autologous stem-cell transplant in patients with Hodgkin’s and non-Hodgkin’s Lymphoma. Bone Marrow Transplant (2003) 31(11):1009–13. 10.1038/sj.bmt.1704050 12774052

[96] Porrata LF Inwards DJ Lacy MQ Markovic SN. Immunomodulation of early engrafted natural killer cells with interleukin-2 and interferon-alpha in autologous stem cell transplantation. Bone Marrow Transplant (2001) 28(7):673–80. 10.1038/sj.bmt.1703203 11704790

[97] Boll B Eltaib F Reiners KS von Tresckow B Tawadros S Simhadri VR Heat shock protein 90 inhibitor BIIB021 (CNF2024) depletes NF-kappaB and sensitizes Hodgkin’s lymphoma cells for natural killer cell-mediated cytotoxicity. Clin Cancer Res (2009) 15(16):5108–16. 10.1158/1078-0432.CCR-09-0213 19671844

[98] Camodeca C Nuti E Tepshi L Boero S Tuccinardi T Stura EA Discovery of a new selective inhibitor of A disintegrin and metalloprotease 10 (ADAM-10) able to reduce the shedding of NKG2D ligands in Hodgkin’s lymphoma cell models. Eur J Med Chem (2016) 111:193–201. 10.1016/j.ejmech.2016.01.053 26871660

[99] Poggi A Zocchi MR. How to exploit stress-related immunity against Hodgkin’s lymphoma: targeting ERp5 and ADAM sheddases. Oncoimmunology (2013) 2(12):e27089. 10.4161/onci.27089 24498565 PMC3894235

[100] Reusch U Burkhardt C Fucek I Le Gall F Le Gall M Hoffmann K A novel tetravalent bispecific TandAb (CD30/CD16A) efficiently recruits NK cells for the lysis of CD30^+^ tumor cells. MAbs (2014) 6(3):728–39. 10.4161/mabs.28591 24670809 PMC4011917

[101] Rothe A Sasse S Topp MS Eichenauer DA Hummel H Reiners KS A phase 1 study of the bispecific anti-CD30/CD16A antibody construct AFM13 in patients with relapsed or refractory Hodgkin lymphoma. Blood (2015) 125(26):4024–31. 10.1182/blood-2014-12-614636 25887777 PMC4528081

[102] Fang F Xiao W Tian Z. NK cell-based immunotherapy for cancer. Semin Immunol (2017) 31:37–54. 10.1016/j.smim.2017.07.009 28838796

[103] Maki G Klingemann HG Martinson JA Tam YK. Factors regulating the cytotoxic activity of the human natural killer cell line, NK-92. J Hematother Stem Cell Res (2001) 10(3):369–83. 10.1089/152581601750288975 11454312

[104] Tonn T Schwabe D Klingemann HG Becker S Esser R Koehl U Treatment of patients with advanced cancer with the natural killer cell line NK-92. Cytotherapy (2013) 15(12):1563–70. 10.1016/j.jcyt.2013.06.017 24094496

[105] Magister S Tseng HC Bui VT Kos J Jewett A. Regulation of split anergy in natural killer cells by inhibition of cathepsins C and H and cystatin F. Oncotarget (2015) 6(26):22310–27. 10.18632/oncotarget.4208 26247631 PMC4673165

[106] Klingemann H Boissel L Toneguzzo F. Natural killer cells for immunotherapy—advantages of the NK-92 cell line over blood NK cells. Front Immunol (2016) 7:91. 10.3389/fimmu.2016.00091 27014270 PMC4789404

[107] Williams BA Law AD Routy B denHollander N Gupta V Wang XH A phase I trial of NK-92 cells for refractory hematological malignancies relapsing after autologous hematopoietic cell transplantation shows safety and evidence of efficacy. Oncotarget (2017) 8(51):89256–68. 10.18632/oncotarget.19204 29179517 PMC5687687

[108] Aleman BM van den Belt-Dusebout AW Klokman WJ Van’t Veer MB Bartelink H van Leeuwen FE. Long-term cause-specific mortality of patients treated for Hodgkin’s disease. J Clin Oncol (2003) 21(18):3431–9. 10.1200/JCO.2003.07.131 12885835

[109] van Nimwegen FA Schaapveld M Janus CP Krol AD Raemaekers JM Kremer LC Risk of diabetes mellitus in long-term survivors of Hodgkin lymphoma. J Clin Oncol (2014) 32(29):3257–63. 10.1200/JCO.2013.54.4379 25154821

[110] Daniels LA Krol AD de Graaf MA Scholte AJ Van’t Veer MB Putter H Screening for coronary artery disease after mediastinal irradiation in Hodgkin lymphoma survivors: phase II study of indication and acceptance dagger. Ann Oncol (2014) 25(6):1198–203. 10.1093/annonc/mdu130 24692582

[111] Behringer K Goergen H Muller H Thielen I Brillant C Kreissl S Cancer-related fatigue in patients with and survivors of Hodgkin lymphoma: the impact on treatment outcome and social reintegration. J Clin Oncol (2016) 34(36):4329–37. 10.1200/JCO.2016.67.7450 27998235

[112] Sud A Thomsen H Sundquist K Houlston RS Hemminki K. Risk of second cancer in Hodgkin lymphoma survivors and influence of family history. J Clin Oncol (2017) 35(14):1584–90. 10.1200/JCO.2016.70.9709 28384078 PMC5455705

